# A Method to Calibrate Angular Positioning Errors Using a Laser Tracker and a Plane Mirror

**DOI:** 10.3390/s25061834

**Published:** 2025-03-15

**Authors:** Bala Muralikrishnan, Meghan Shilling, Vincent Lee, Olga Ridzel, Glenn Holland, John Villarrubia

**Affiliations:** 1 Sensor Science Division, National Institute of Standards and Technology, Gaithersburg, MD 20899, USA; katharine.shilling@nist.gov (M.S.); vincent.d.lee@nist.gov (V.L.); 2 Microsystems and Nanotechnology Division, National Institute of Standards and Technology, Gaithersburg, MD 20899, USA; olga.ridzel@nist.gov (O.R.); glenn.holland@nist.gov (G.H.); john.villarrubia@nist.gov (J.V.); 3 Theiss Research, La Jolla, CA 92037, USA

**Keywords:** angular positioning error, laser tracker, rotation stage

## Abstract

We describe a method to calibrate angular positioning errors of a rotation stage using a laser tracker (LT), a plane mirror mounted on the stage, and stationary registration nests placed around the stage. Our technique involves determining the direction of the normal vector to the plane of the mirror at each angular step by performing two LT measurements—one directly to a stationary spherically mounted retroreflector (SMR), and another to the same SMR by bouncing the laser off a mirror mounted on the rotation stage. Because the angular range that can be measured from a single LT station is limited by the angle of incidence on the mirror, multiple LT stations are necessary to cover the full 360°, hence the need for stationary registration nests to tie the LT data into a common coordinate frame. We compare this technique against a direct approach involving a rigid bar with two SMRs mounted on the rotation stage so that we can measure the direction of the line joining the SMRs at each angular position using the LT and, therefore, the angle between positions. Through experiments, we demonstrate that our mirror-based approach provides errors on the order of ±0.5″, smaller than the ±1.5″ for the direct approach, when compared against a reference instrument with accuracy better than 0.3″. Through simulations, we estimate the uncertainty in our mirror-based angle measurements to be 0.4″ (*k* = 2). Placing the LT close to and the SMR away from the rotation stage results in lower uncertainty for our mirror-based angle measurements.

## 1. Introduction

There are six error motions associated with an axis of rotation, three angular and three translational, that are a function of the angular position about the axis. Terminology and description on these errors can be found in the ASME B89.3.4 [1] and ISO 230-7 standards [2]. Characterizing all error motions associated with an axis of rotation is particularly important in precision machining and positioning applications (see [3,4] for a review of the methods).

For many industrial and scientific applications, it is sufficient to calibrate just the angular positioning error (ECC in [2]) about the axis of rotation. The general problem of angle sensing is discussed in [5,6]. In this paper, we are concerned with the practical problem of calibrating the angular positioning errors in situ without modifications to the underlying hardware.

Angular positioning errors can be measured at a fraction of an arcsecond accuracy using an autocollimator if the rotation stage is repeatable and can accommodate a polygon or a nulling autocollimator with a plane mirror [7,8,9,10,11,12]. Interferometry-based solutions may also be used if the table can accommodate the reflectors [13,14]. One commercially available interferometry-based solution [13] has a stated accuracy of 1″. An optical approach using a 1D grating, two position-sensitive detectors, and a laser diode is presented in [15]. In this approach, the laser beam from the laser diode strikes the grating and the resulting diffraction patterns are sensed by two PSDs, enabling the calculation of the angular positioning error with an uncertainty of 1.5″. A camera-based approach is described in [16], where a plate with lines is imaged using a camera, and circle closure is used to estimate the angular positioning error. A low-accuracy but practical camera-based approach is presented in [17], where a checkerboard target mounted on the table is imaged to detect angles with an accuracy of 0.1°.

Angular positioning errors of rotation stages integrated into coordinate measuring machines (CMMs) can be measured using a ball plate [18,19] or a hole plate [20] by probing the features at multiple angular positions and using error separation techniques. Angular positioning errors in the spindles of machine tools are mapped using a double-ball bar [21] or a touch-trigger probe mounted on the spindle and artifacts mounted on the table of the machine [22]. There is also literature on the use of laser trackers (LTs) and laser tracers for estimating the errors of rotation stages. The errors of a rotation stage mounted on a CMM are calibrated using a single laser tracer mounted on the rotation stage [23,24] and a cat-eye reflector mounted on the ram of the CMM or the machine tool. At each angular position of the rotation stage, the reflector is commanded to several positions, and the distance to each position is recorded. An optimization process is used to compute the angular positioning errors from the LT/tracer. A different scheme is reported in [24,25], where multiple laser tracers are placed on the table of a machine tool while one or more reflectors are mounted on the spindle. The tracers record the distances to the targets at each angular position, and the errors are computed from those data. A modification is proposed in [26,27], where a single LT or tracer measures the distance to multiple targets on a rotation table. A method to map the angular positioning errors of a robot by measuring the distance from the end effector using an interferometer to fixed retroreflectors on the table is described in [28].

As is evident from the review, there are a variety of techniques reported in the literature for the in-situ measurement of angular positioning errors. Almost all techniques require the mounting of large or heavy hardware on the rotation stage, except perhaps the case of the nulling autocollimator, which only requires a small mirror mounted on the stage. There are applications where the rotation stage can accommodate a small mirror but there is not enough room for an autocollimator to function effectively because of insufficient return light. The retarding field analyzer (RFA) experiment [29,30] currently being pursued here at NIST is an example of such an application. The experiment involves bombarding a sample mounted on a stepper motor with electrons from an electron gun. As the sample is positioned at different angles (at a desired accuracy of 0.1°), the secondary electrons are measured and compared against published models [30]. The physical constraint imposed by the setup is the motivation for the development of the technique reported in this paper. We use an LT, a plane mirror mounted on the stepper motor shaft, and some LT registration nests in a simple yet elegant manner to map the angular errors over the full circle.

The rest of the paper is organized as follows. We describe the approach in Section 2, experiments and results in Section 3, a discussion on uncertainty and sensitivity to system parameters in Section 4, a brief description of the RFA setup in Section 5, and conclusions in Section 6.

## 2. Approach

**Figure 1:**
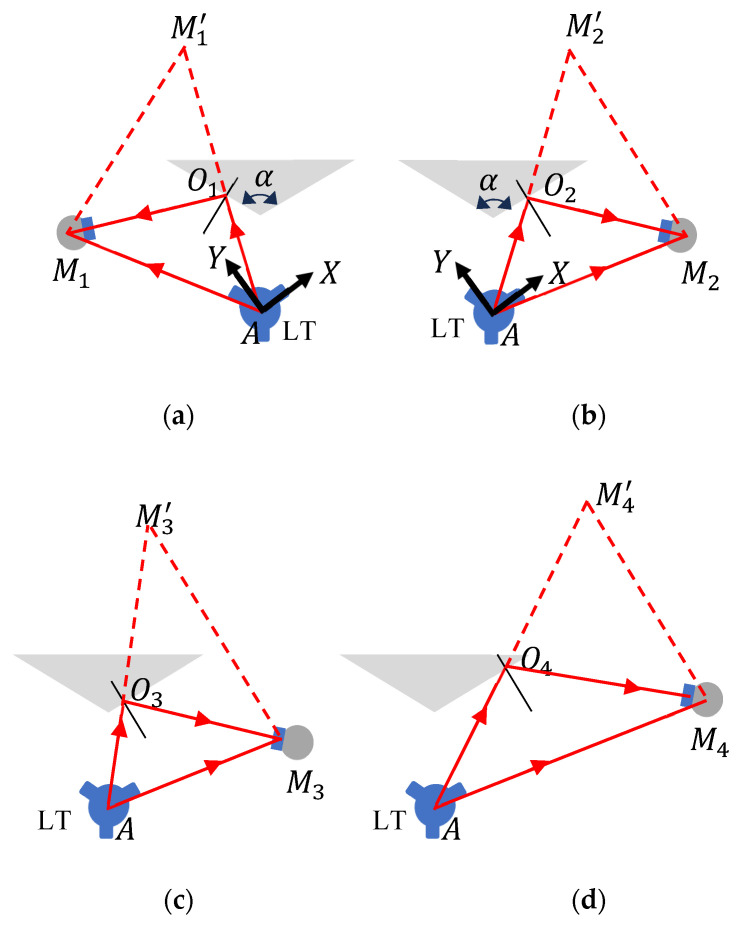
Measuring the direction of the normal vector through two measurements, one direct and one indirect (after reflection at the surface), for (**a**) one face of the wedge and (**b**) the other face of the wedge. The top view is shown. Parts (**b**–**d**) show the measurement of the normal vectors when the laser strikes the face at different points (at $O_{2}$, $O_{3}$, and $O_{4}$, respectively). The normal vectors are all parallel, thus, there is no requirement for the laser to strike a specific point on the face.

The basic idea behind our angle measurement approach is illustrated in Figure 1. Consider a wedge (shown by the shaded triangle) placed in front of an LT, as shown in Figure 1. Suppose we are interested in measuring the angle α between two faces of this wedge. We first determine the direction of the normal vector at $O_{1}$ on one face of the wedge, by making two measurements with the LT—a direct measurement to a spherically mounted retroreflector (SMR) at $M_{1}$ and an indirect measurement to the same SMR after reflection off the surface at $O_{1}$ (see Figure 1a). Because the second measurement appears at $M_{1}^{\prime }\;$ in the LT coordinate system, from basic geometry, the normal vector to the surface at $O_{1}$ is parallel to $\vec{M_{1}A}-\vec{M_{1}^{\prime }A\;}$, i.e., the difference in the coordinates of $M_{1}$ and $M_{1}^{\prime }$ (because A is the origin of the LT coordinate system). The unit normal vector can then be given by:

(1) $$\vec{n_{1}}=\frac{\vec{M_{1}A}-\vec{M_{1}^{\prime }A}}{\left|\vec{M_{1}A}-\vec{M_{1}^{\prime }A}\right|}=\frac{\vec{M_{1}M_{1}^{\prime }}}{\left|\vec{M_{1}M_{1}^{\prime }}\right|}$$

Without disturbing the LT and the wedge, we perform the same measurement at the other face of the wedge (see Figure 1b). The normal vector to the surface at $O_{2}$ is parallel to $\vec{M_{2}A}-\vec{M_{2}^{\prime }A}$. The unit normal vector is given by:

(2) $$\vec{n_{2}}=\frac{\vec{M_{2}A}-\vec{M_{2}^{\prime }A}}{\left|\vec{M_{2}A}-\vec{M_{2}^{\prime }A}\right|}=\frac{\vec{M_{2}M_{2}^{\prime }}}{\left|\vec{M_{2}M_{2}^{\prime }}\right|}$$

The angle α can be estimated from the dot product of the two vectors as:

(3) $$\alpha =\cos^{-1}\left(\vec{n_{1}}.\vec{n_{2}}\right)$$

A significant advantage of our technique is that the LT laser can bounce off from any position on the face of the wedge. Figure 1b–d show the laser striking the face at $O_{2}$, $O_{3}$, and $O_{4}$, respectively. The SMR positions $M_{2}$, $M_{3}$, and $M_{4}$ are different, but the normal vector directions $\vec{M_{2}M_{2}^{\prime }}$, $\vec{M_{3}M_{3}^{\prime }}$, and $\vec{M_{4}M_{4}^{\prime }}$ are all parallel. This allows the measurement of the angle between faces without the need to carefully direct the laser to a specific spot on the surface, assuming, of course, that the surface is flat to a level that does not affect the accuracy needs of the angle measurement. While the approach presented here can be extended, in principle, to the measurement of angular positioning errors of a rotation stage using a mirror, there are some subtilities to consider, as explained in Section 2.2.

At a first glance, our technique might appear to be limited by the angular accuracy of the LT. However, there are simple steps, based on our understanding of the error model of an LT [31], that can be taken to reduce the influence of systematic errors. For instance, we averaged the front- and back-face coordinates for each measurement of the SMR, thus eliminating most sources of systematic error. We can also adopt the four-orientation method described in [32] to further reduce systematic errors in the angle measurement. While we employed the front-face back-face averaging, we did not perform the four-orientation approach in this case because the angular accuracy needs of the RFA experiment are not stringent.

In addition to the mirror-based approach, we also considered a direct approach, where we measured the direction of a bar mounted rigidly on the table. We describe that approach in Section 2.1 and then describe the mirror-based approach in Section 2.2. In our discussion, we consider the measurement of angular positioning errors in increments of 22.5° over a full circle.

### 2.1. Direct Approach

**Figure 2:**
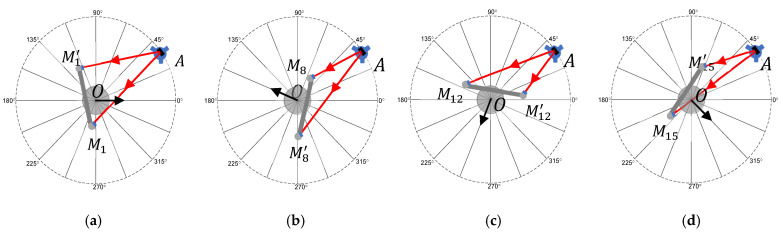
Direct approach with the LT located at A, center of rotation at O, and a bar with two SMRs (at $M_{i}$ and $M_{i}^{\prime }$) rigidly mounted on the rotation stage. Encoder reading (**a**) 0°, (**b**) 157.5°, (**c**) 247.5°, and (**d**) 315°. The top view is shown.

We considered a bar with two 38.1 mm (1.5 inch) SMR nests (located at $M_{1}$ and $M_{1}^{\prime }$) mounted rigidly on the rotation stage, as shown in Figure 2a. Note that $M_{1}^{\prime }$ represented a virtual point in the previous section but denotes a physical point here. We used the same notation to ensure consistency in the equations. There were no restrictions on the placement of the bar on the table. The LT was placed near the rotation stage and at the height of the table. The SMRs were placed facing the LT. Suppose that the encoder was reading 0° at this position (indicated by the black arrow in the figure). We measured the center of each SMR by averaging data from both faces of the LT. We calculated the vector $\vec{M_{1}M_{1}^{\prime }}=\vec{M_{1}A}-\vec{M_{1}^{\prime }A\;}$, i.e., the difference in the coordinates of $M_{1}$ and $M_{1}^{\prime }$ (because A is the origin of the LT coordinate system). Subscript 1 indicates the first angular position of the rotation stage. We calculated the unit vector $\vec{n_{1}}$, as shown in Equation (1). We then rotated the table to the next angular position, re-oriented the SMRs to face the LT, and repeated the previous steps to calculate the unit vector $\vec{n_{2}}$. We repeated this process for each angular position *I* and calculated the normal vector $\vec{n_{i}}$. Figure 2b–d show a few positions of the rotation stage.

If the SMRs at the ends of the bar are at the same height from the table, then the dot product between the unit vectors at the *i*th angular position $\vec{n_{i}}$ and the first position $\vec{n_{1}}$ will provide the angle between those positions. It is, however, practically challenging to ensure this condition. In the more realistic case, where they are at different heights, we had to project the unit vectors $\vec{n_{i}}$ to the plane of rotation before we could calculate the angles. This plane can be easily determined from the center coordinates of one of the SMRs over the full circle through a least-squares fitting process. After projecting the vectors to the plane, we could calculate the angle at any position *I* with respect to the starting position (*I* = 1) from the dot products of the corresponding vectors. Because we calculated the angle in the plane of rotation, it was not necessary for the plane of rotation to be parallel to the XY plane of the LT coordinate system. Thus, there were no special alignment requirements for the measurement.

The advantage of the direct approach was that we could calibrate the angle errors over the full 360° range from a single position of the LT. There were no significant restrictions on the placement of the bar or the LT, other than the LT being at approximately the same height as the table (to reduce the effect of LT systematic errors [31,32]). The disadvantage was the need to orient the SMRs toward the LT for each angular step. Further, some rotation stages, especially smaller ones, might not accommodate a bar with two SMRs, which are necessary for calibration, as is the case for the RFA experiment.

### 2.2. Mirror-Based Approach

**Figure 3:**
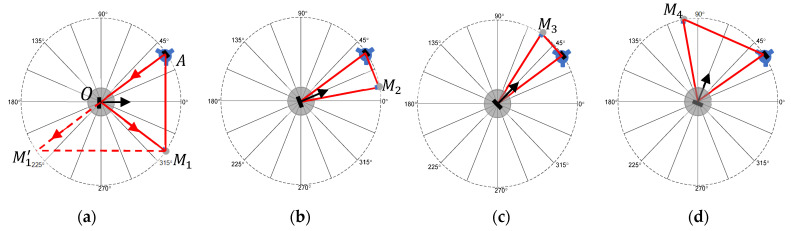
Mirror-based approach with the LT located at A, center of rotation at O, and a mirror (solid black rectangle) mounted on the rotation table. Angular positions (i.e., mirror normal vector pointing along) (**a**) 0°, (**b**) 22.5°, (**c**) 45°, and (**d**) 67.5°. The top view is shown.

We considered a plane mirror mounted on the rotation stage, as shown in Figure 3a, with the LT at A. In the figure, the normal vector to the mirror (solid black rectangle in the figure) is along the 0° encoder position of the rotation stage. A stationary nest is located at $M_{1}$. We performed two measurements at this position—one was a direct measurement of SMR at $M_{1}$ and the other was an indirect measurement of the same SMR, i.e., after reflecting off the mirror on the rotation stage. In the LT coordinate system, the second measurement appeared to be at $M_{1}^{\prime }$. We then rotated the table to the next angular position, moved the stationary nest, and repeated the previous steps. Measurements at the three remaining angular positions, 22.5°, 45°, and 67.5°, are shown in Figure 3b–d.

The advantage of this approach was that there were no restrictions on the placement of the plane mirror on the table or for the alignment of the plane of rotation with respect to the LT. Further, the laser could strike the mirror at any point on the surface (assuming the mirror is flat to a level that it does not affect angle measurements), as mentioned earlier in Section 2, so there were no burdensome alignment or setup restrictions. However, because the LT laser must bounce off the mirror, the angular range that can be measured from a single LT station was limited by the angle of incidence on the mirror (theoretically ±90°, but practically less than about ±50°). Thus, multiple LT stations were required to cover the full 360°. There are two ways to tie data together from the multiple LT stations, stitching and registration, and that affected how we processed the data. These are discussed next.

#### 2.2.1. Stitching

**Figure 4:**
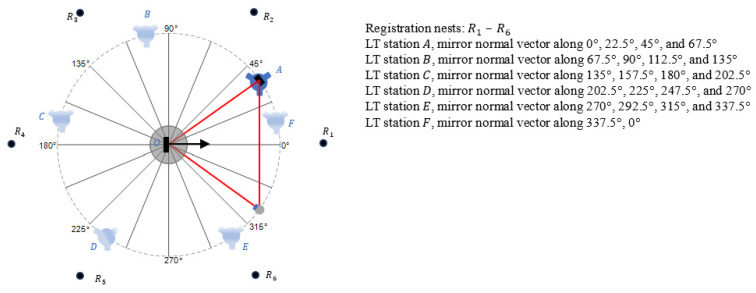
Setup consisting of six LT stations and six registration nests.

Figure 4 shows the measurement of angular positioning errors of a rotation stage in steps of 22.5° from six stations of the LT, A−F. The commanded angles of the rotation stage are given by:

(4) $$\theta_{i}=(i-1)\frac{2\pi }{16},$$

where *i* is the angular position index. While 16 positions covered the full circle (when measurements were made in increments of 22.5°), we measured errors at 17 positions so that we had an overlapping position (at 0°) to assess closure errors.

Measurements were performed from LT station A for angular positions (i.e., mirror normal vector pointing along) 0°, 22.5°, 45°, and 67.5°, as described earlier. When the LT was moved to the next station, measurements were repeated at the last angular step of the previous LT station; thus, measurements from LT station B were performed for angular positions 67.5°, 90°, 112.5°, and 135°. For the final LT station, F, measurements were performed for angular positions 337.5° and 0°.

**Figure 5:**
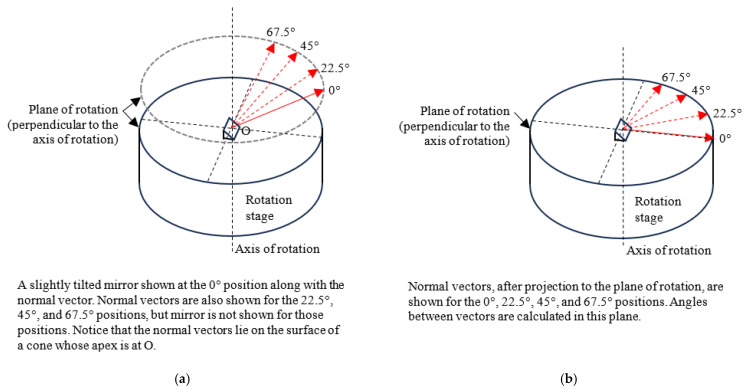
(**a**) Showing the normal vectors of a slightly tilted mirror at angular positions 0°, 22.5°, 45°, and 67.5°, and (**b**) mirror normal vectors after projection to the plane of rotation. Angles are calculated in this plane.

From the data acquired with the LT at station A, we calculated the unit normal vectors $\vec{n_{1,A}},\;\vec{n_{2,A}},\;\vec{n_{3,A}},\;and\;\vec{n_{4,A}}$ at angular positions 0°, 22.5°, 45°, and 67.5°, respectively. Subscript A indicates that the measurements were made from LT station A. It is unlikely that the mirror was positioned in such a manner that the normal vector to the mirror was parallel to the plane of rotation. We must, therefore, project these normal vectors to the plane of rotation before calculating angles. However, unlike in the direct approach case, we did not have data in the form of SMR centers to fit a least-squares plane. However, if we visualize the normal vectors so that one end is coincident, the vectors lie on the surface of a cone (assuming the tilt errors of the rotation stage are negligible) so that the other end lies on a circle (see Figure 5a). We could, therefore, fit a least-squares plane to these end points and project the normal vectors to that plane (see Figure 5b). This idea is also applicable to the direct approach case but we did not apply it there because we had SMR centers over the full circle that we could more easily use to find the plane. We normalized the vectors after projection and calculated the angles for position *i* = 1–4 with respect to 1. We let the unit normal vectors after projection to the plane of rotation corresponding to angular positions 0°, 22.5°, 45°, and 67.5° be represented using the same variables for convenience, i.e., $\vec{n_{1,A}},\;\vec{n_{2,A}},\;\vec{n_{3,A}},\;and\;\vec{n_{4,A}},$ respectively. We will not be referring to the normal vectors prior to projection in subsequent discussions in this section; therefore, there is not a need to create a new variable.

The angles and errors are then given by:

(5) $$\alpha_{i,A}=\cos^{-1}\left(\vec{n_{i,A}}.\vec{n_{1,A}}\right),\;i=1\;to\;4$$

(6) $$e_{i}=e_{i,A}=\theta_{i}-\alpha_{i,A},\;i=1\;to\;4$$

From the data acquired with the LT at station B, we calculated the unit normal vectors $\vec{n_{4,B}},\;\vec{n_{5,B}},\;\vec{n_{6,B}},\;and\;\vec{n_{7,B}},$ corresponding to angular positions 67.5°, 90°, 112.5°, and 135°, respectively. We fit a least-squares plane, projected the normal vectors to that plane, and normalized the vectors. We calculated the angles and the errors for position *i* = 5–7 with respect to 4 (corresponding to the 67.5° angular position):

(7) $$\alpha_{i,B}=\cos^{-1}\left(\vec{n_{i,B}}.\vec{n_{4,B}}\right)$$

(8) $$e_{i,B}={{(\theta }_{i}-\theta_{4})-\alpha_{i,B}=\theta }_{i-4}-\alpha_{i,B},\;i=5\;to\;7$$

Note that in Equation (8), the commanded angle for positions 5, 6, and 7 must be determined with respect to position 4, hence the term ${(\theta }_{i}-\theta_{4}),$ which is equivalent to $\theta_{i-4}$. The error with respect to the first position of the LT station A was then obtained by summing the error of the last position of the previous station:

(9) $$e_{i}=e_{i,B}+e_{4,A},\;i=5\;to\;7$$

We repeated this process for the remaining LT stations, yielding the following equations:

(10) $$\alpha_{i,C}=\cos^{-1}\left(\vec{n_{i,C}}.\vec{n_{7,C}}\right)$$

(11) $$e_{i,C}=\theta_{i-7}-\alpha_{i,C},\;i=8\;to\;10$$

(12) $$e_{i}=e_{i,C}+e_{4,A}+e_{7,B},\;i=8\;to\;10$$

(13) $$\alpha_{i,D}=\cos^{-1}\left(\vec{n_{i,D}}.\vec{n_{10,D}}\right)$$

(14) $$e_{i,D}=\theta_{i-10}-\alpha_{i,D},\;i=11\;to\;13$$

(15) $$e_{i}=e_{i,D}+e_{4,A}+e_{7,B}+e_{10,C},\;i=11\;to\;13$$

(16) $$\alpha_{i,E}=\cos^{-1}\left(\vec{n_{i,E}}.\vec{n_{13,E}}\right)$$

(17) $$e_{i,E}=\theta_{i-13}-\alpha_{i,E},\;i=14\;to\;16$$

(18) $$e_{i}=e_{i,E}+e_{4,A}+e_{7,B}+e_{10,C}+e_{13,D},\;i=14\;to\;16$$

(19) $$\alpha_{i,F}=\cos^{-1}\left(\vec{n_{i,F}}.\vec{n_{16,F}}\right)$$

(20) $$e_{i,F}=\theta_{i-16}-\alpha_{i,F},\;i=17$$

(21) $$e_{i}=e_{i,F}+e_{4,A}+e_{7,B}+e_{10,C}+e_{13,D}+e_{16,E},\;i=17$$

#### 2.2.2. Registration

With the LT at station A, we measured the SMR nests at each of the four angular positions, 0°, 22.5°, 45°, and 67.5°. We calculated the corresponding unit normal vectors $\vec{n_{1,A}},\;\vec{n_{2,A}},\;\vec{n_{3,A}},\;and\;\vec{n_{4,A}}$. We also measured the six registration nests, $R_{1}–\;R_{6}$. While three nests were sufficient, choosing a larger number of nests helped to minimize the effect of LT errors in the registration process. Our choice of six nests was dictated by the stands and SMRs we had available for use.

We moved the LT to station B and measured the SMR nests at each of the four angular positions—67.5°, 90°, 112.5°, and 135°. We calculated the corresponding unit normal vectors $\vec{n_{4,B}},\;\vec{n_{5,B}},\;\vec{n_{6,B}},\;and\;\vec{n_{7,B}}.$ We measured the six registration nests and calculated the matrix to transform the data acquired from LT station B to A. Using this matrix, we transformed the normal vectors to the frame of the LT at station A. Let the resulting normal vectors be $\vec{n_{4,B,A}},\;\vec{n_{5,B,A}},\;\vec{n_{6,B,A}},\;and\;\vec{n_{7,B,A}}$, where the subscript B indicates that the data were acquired from station B and the subscript A indicates that the data had since been transformed to station A. We repeated this process for the other LT stations and calculated the normal vectors.

As we noted earlier, it is unlikely that the mirror was positioned in such a manner that the normal vectors to the mirror were parallel to the plane of rotation. We projected these normal vectors to the plane of rotation, as described in Section 2.2.1, normalized them, and calculated angles with respect to the 0° position. Note that in this case, we collected the normal vectors from all the LT positions, transformed them to LT station *A*, and then fit a single plane. In the case of stitching, we had to perform this operation for each LT station. Also, as in Section 2.2.1, we used the same variables for the normal vectors after projection to the plane for convenience. We will henceforth not be referring to the normal vectors prior to projection in this section.

The angles are given by:

(22) $$\alpha_{i}=\cos^{-1}\left(\vec{n_{i,A}}.\vec{n_{1,A}}\right),\;i=1\;to\;4\;$$

(23) $$\alpha_{i}=\cos^{-1}\left(\vec{n_{i,B,A}}.\vec{n_{1,A}}\right),\;i=4\;to\;7$$

(24) $$\alpha_{i}=\cos^{-1}\left(\vec{n_{i,C,A}}.\vec{n_{1,A}}\right),\;i=7\;to\;10\;$$

(25) $$\alpha_{i}=\cos^{-1}\left(\vec{n_{i,D,A}}.\vec{n_{1,A}}\right),\;i=10\;to\;13\;$$

(26) $$\alpha_{i}=\cos^{-1}\left(\vec{n_{i,E,A}}.\vec{n_{1,A}}\right),\;i=13\;to\;16\;$$

(27) $$\alpha_{i}=\cos^{-1}\left(\vec{n_{i,F,A}}.\vec{n_{1,A}}\right),\;i=16\;to\;17\;$$

We then calculated the errors in the angles:

(28) $$e_{i}=\theta_{i}-\alpha_{i},\;i=1\;to\;17$$

Because we measured some angular steps twice, i.e., as the last measurement from a given LT station and as the first measurement from the next LT station, we had repeat measurements for those positions. These repeat measurements are not required for the registration approach but are required for the stitching approach.

## 3. Experiments and Results

We first demonstrate the application of this technique using a calibrated angle polygon and then discuss the experiments performed and results obtained using a precision air-bearing spindle. Use of this technique on the RFA experimental setup will be presented in a future paper. The LT used in the experiments had an angular accuracy specification of ±15 µm + 6 µm/m, distance accuracy of 0.5 µm/m, and a dynamic lock on specification of 10 µm.

### 3.1. Polygon Measurements

**Figure 6:**
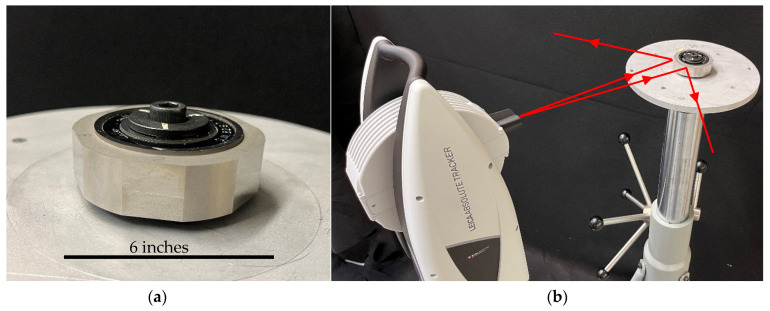
(**a**) The polygon used for the validation experiment, and (**b**) showing the placement of the LT and the polygon.

We measured two faces of a calibrated angle polygon and calculated the angle between those faces as a validation of this technique. The angle polygon is shown in Figure 6a. The nominal angle between faces was 60°. Figure 6b shows the placement of the LT in front of the polygon. The stationary SMRs on either side of the polygon are not shown. A script was written to repeatedly measure points $M_{1},\;M_{1}^{\prime }$, $M_{2},$ and $M_{2}^{\prime }\;$ (in Figure 1) over a period of 8 h, resulting in a total of 360 points (90 points each) from which 90 angles were computed. The calibrated deviation from nominal (60°), obtained from autocollimator measurements made at NIST, was +0.54″, with an uncertainty (*k* = 2) of 0.2″. The mean of the calculated angles from the 90 LT measurements was 0.38″ more than the nominal value of 60°, for an error of −0.16″. The standard deviation of the 90 measurements was 0.17″. If the only source of uncertainty is the repeatability of the LT, the uncertainty in the mean should be about $\frac{0.17}{\sqrt{90}}=0.02$″. Clearly, the observed error (magnitude only) of 0.16″ is significantly larger in comparison to this value, indicating the presence of other systematic error sources that were not attenuated by averaging. See Section 4 for a further discussion on uncertainty.

There are at least two sources of systematic error that we have not accounted for. The first is the inherent systematic errors in our LT that varied as a function of the azimuth angle. While we did average front and back data, there were some errors that were not eliminated by this process. The effect of these errors can be further attenuated through the four-orientation method. We have not done that yet, as our application does not require such a high accuracy. The other error source was due to the difference between the autocollimator and the LT, in that the autocollimator measures over the full face of the polygon while the LT only measures over a small region. The flatness of the face can produce errors on the order of a few tenths of a second [33,34].

### 3.2. Precision Spindle Measurements

The primary focus of this paper was the measurement of the angular positioning errors of a rotation stage. We demonstrate the application of our technique using a precision air-bearing spindle and an LT in this section.

#### 3.2.1. Verification of Spindle Performance

**Figure 7:**
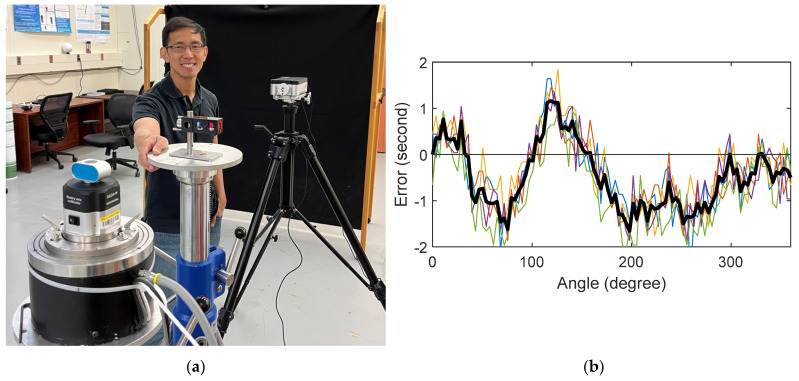
(**a**) Setup showing the calibration of the angle errors using the XR-20 on the spindle and the angular optics and laser head on stands near the spindle. (**b**) Difference between the angles recorded by the XR-20 and the commanded position of the spindle. Note that these are ‘corrections’, not ‘errors’, because the reference instrument is the XR-20. The errors in the spindle would be equal in magnitude but opposite in sign. The Y axis is labeled ‘errors’, not ‘corrections’, to be consistent with later plots, which are in fact errors. The different colors represent measurement runs on different days. Solid black line is the average.

The air-bearing spindle has an encoder with 94,400 counts, i.e., 3.8 millidegrees per count. To determine the angular positioning accuracy of the spindle, we first compared the spindle against a Renishaw XR-20 rotary axis calibration equipment (hereafter simply referred to as the XR-20) that has a stated accuracy of 1″. However, the calibration report accompanying the instrument indicates a maximum error of 0.2″ with an uncertainty of 0.3″. Figure 7a shows the XR-20 mounted on the spindle, and the angular optics and laser head mounted on stands next to the spindle. We measured the angular errors in steps of 3.6° over the full circle. We repeated this measurement several times over multiple days. Figure 7b shows the difference between the angles recorded by the XR-20 and the commanded position of the spindle over several runs obtained on different days. We considered the average (shown by the solid black line in the figure) as the reference against which we compared the measurements obtained using the direct and mirror-based approaches. The measurements over several days lay within a ± 1″ interval about the mean.

#### 3.2.2. Direct and Mirror-Based Approaches

**Figure 8:**
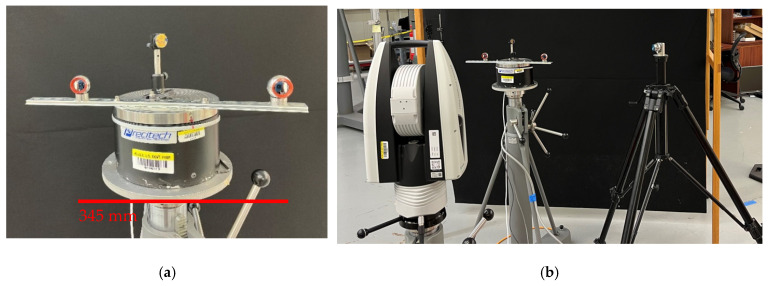
(**a**) Bar mounted on the spindle with two SMRs for angle measurement using the direct approach, and a plane mirror centrally mounted for the angle measurement using the mirror-based approach. (**b**) Showing the LT, the spindle, and the stationary SMR on a tripod for the mirror-based approach.

We mounted a bar with two SMR nests on the spindle, as shown in Figure 8a. The distance between the nests was about 345 mm. The bar and nests were mounted in an arbitrary manner, resulting in one nest being closer to the center than the other. We also mounted a plane mirror near the center of the spindle so that the axis of rotation was close to and centered on the face of the mirror (to the extent possible without special alignment tools). The experiments were performed as follows. The LT was stationed at A (see Figure 4), as close to the spindle as possible. We first performed the direct measurements using the LT and SMRs mounted on the bar on the spindle. That is, with the SMRs pointing toward the LT, we measured the center of both SMRs by averaging the front- and back-face data. We then indexed the spindle by 22.5° and repeated the previous steps. We continued this process until we completed a full circle and re-measured the 0° position for closure.

We then performed the mirror-based measurements using the LT, the plane mirror on the spindle, and another SMR located on a tripod, as shown in Figure 8b. With the LT at station A, we first measured the six registration nests (again, as the average of front- and back-face data). We then measured the center of the SMR on the tripod directly and indirectly by bouncing the laser off the mirror. We indexed the spindle by 22.5° and repeated the previous step. Note that the tripod must be re-positioned so that we can capture the laser beam reflecting off the mirror. For the LT in station A, we performed these measurements at angular positions of 0°, 22.5°, 45°, and 67.5°. We then moved the LT to station B and measured the six registration nests. We then measured the SMR on the tripod (direct as well as off the mirror) at angular positions of 67.5°, 90°, 112.5°, and 135°. We repeated this process for the remaining LT stations according to Figure 4.

**Figure 9:**
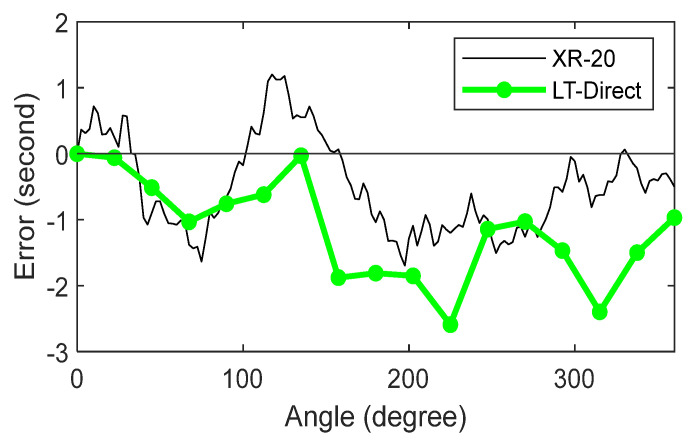
XR-20 and LT direct approach data, prior to correcting for closure error.

Next, we present the results from the direct measurements. Figure 9 shows the XR-20 and LT data that have not yet been corrected for closure errors, i.e., the error in the measurement of the 0° position at the end of the run. Ideally, this value should be zero, but it was not, for both the XR-20 and the LT data. The closure error for the XR-20 was about 0.5″, and about 1″ for the direct approach. This closure error was not only due to drift in the measurements but also due to the positioning error in the spindle itself.

**Figure 10:**
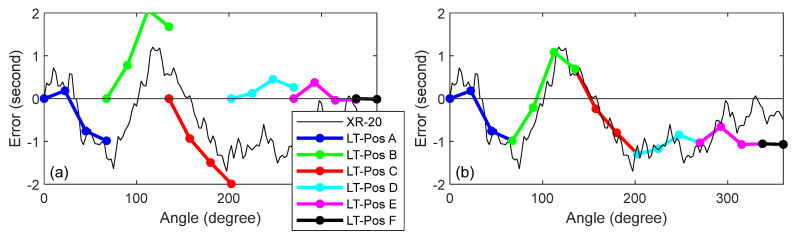
(**a**) XR-20 and LT mirror-based approach before stitching, and (**b**) XR-20 and LT mirror-based approach after stitching.

We present the results for the mirror-based approach before presenting the results after correcting for closure error. Figure 10a shows the XR-20 and LT data collected from the six stations for the mirror-based stitching approach. Because we did not use registration nests, the error in the first angular step at each of the LT stations was zero. Figure 10b shows the errors after stitching, i.e., after stacking the errors from each LT position on top of the errors from the previous position. The closure error was about 1″.

**Figure 11:**
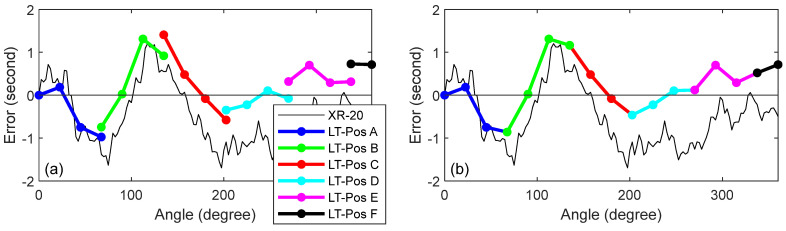
(**a**) XR-20 and LT mirror-based approach before averaging repeats, and (**b**) XR-20 and LT mirror-based approach after averaging repeats.

Figure 11a shows the XR-20 and LT data collected from the six stations for the mirror-based registration approach. Every fourth angular step was measured twice, once as the last measurement from a given LT position, and then as the first measurement from the next LT position. This can be seen in Figure 11a, where there are small offsets, on the order of 0.5″, between LT stations. We averaged those repeated measurements, and the results are shown in Figure 11b. The closure error was about 0.8″.

**Figure 12:**
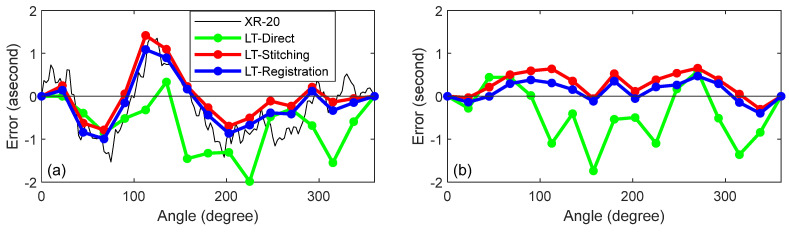
(**a**) XR-20, LT direct approach, and mirror-based approach by stitching and registration, and (**b**) the difference between the LT and XR-20 data.

To remove the closure error, we assumed uniform drift and subtracted the slope. The resulting corrected data are shown in Figure 12a for the XR-20 data, LT direct approach, and the LT mirror-based stitching and registration approaches. Notice that the errors at the 360° position are now zero for all cases. Figure 12b shows the difference between the LT and the XR-20 data. This is the error in the LT measurements when compared against the reference instrument, the XR-20. In the case of the direct approach, the errors were large, about 1.5″, but the errors were less than about 0.5″ for the mirror-based approaches.

## 4. Uncertainty and Sensitivity

We first discuss the uncertainty in our angle measurements for the direct and mirror-based approaches and then discuss the sensitivity of setup parameters (such as distance of the LT from the axis of rotation) to the uncertainty.

**Figure 13:**
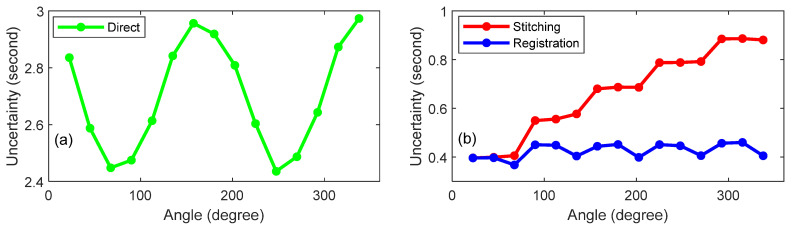
Uncertainty (*k* = 2) as a function of the angle for (**a**) the direct approach and (**b**) the mirror-based approach.

We assessed the uncertainty in the angle using a Monte Carlo simulation (MCS) by propagating the uncertainty in the range and angles of the LT. The range and angle uncertainties of the LT can be assessed from two sets of measurements we have performed. From the measurements made on the polygon over a period of 8 h, we calculated the one standard deviation repeatability of the horizontal angle, the vertical angle, and the range. These were 0.18″, 0.28″, and 0.7 µm, respectively. From the data collected on the six registration nests from the six LT stations, we performed a bundle adjustment [35] and calculated the standard deviation of the residuals. These were 0.18″, 0.14″, and 0.7 µm. We took the larger of the values (0.18″ for the horizontal angle, 0.28″ for the vertical angle, and 0.7 µm for range) and performed an MCS for the specific conditions of the direct approach experiment. The resulting uncertainties (*k* = 2) as a function of the angle are showin in Figure 13a. The periodicity in the plot is because of the geometry of the setup, the direction of the uncertainty vector (especially of the horizontal angle), and how they all combined to affect the result. The amplitude of the periodicity is small, about 0.5″, in comparison to the mean uncertainty, which is about 2.7″.

We then performed an MCS for the specific conditions of the mirror-based experiment. The resulting uncertainties (*k* = 2) as a function of the angle are shown in Figure 13b. Note that the scale of figures for parts (a) and (b) are different. The angle uncertainties for the direct approach are larger than those for the mirror-based approach by a factor of at least four. For the mirror-based approach, registration provided the same low uncertainties for all angles, about 0.4″. In the case of stitching, the uncertainties stacked as we stitched errors, hence the increasingly larger uncertainties of 0.4″ to 0.9″, from 0° to 360°.

To assess whether the estimates of uncertainty were reasonable, we compared the uncertainties against the observed errors. The uncertainties and errors are shown in Table 1. In the case of the direct approach, the errors were all smaller than the uncertainty. For the mirror-based approach, the uncertainty estimates and observed errors were almost equal, indicating that our estimate of uncertainty was optimistic. That was to be expected because all sources of systematic error were not accounted for when calculating the uncertainty in the LT range and angles. A more rigorous uncertainty budget is a future task.

**Figure 14:**
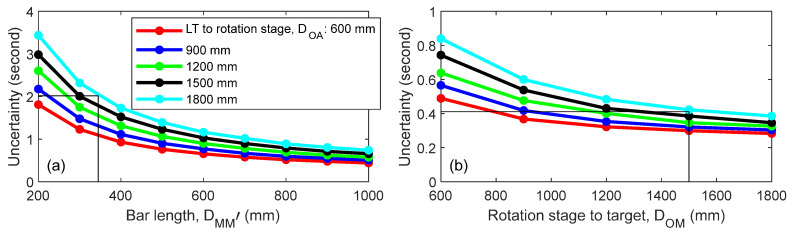
Sensitivity to setup parameters, i.e., the uncertainty (*k* = 2) in the angle for a variety of cases for (**a**) the direct approach and (**b**) the mirror-based approach.

We now address the sensitivity of the setup parameters to the uncertainty in the angle. In the case of the direct approach, there were two parameters we could change—the distance of the LT to the rotation stage (*D_OA_*) and the length of the bar, i.e., the distance between the nests (*D_MM_*_′_; see Figure 2). For this discussion, we assumed that the nests were equidistant from the center. Using the same values for uncertainty in the range and angles as discussed earlier, we calculated the uncertainty (*k* = 2) in the angle through MCS for a variety of cases and plotted the results in Figure 14a. Decreasing the distance of the LT to the rotation stage and increasing the distance between the nests resulted in a smaller angle uncertainty. The solid black vertical and horizontal lines in the figure capture the situation encountered in our experiment, where *D_MM_*_′_ = 345 mm, *D_OA_* = 1800 mm, and uncertainty was 2″. This value is slightly different from the value reported in Table 1 because the simulations were performed for the idealized case, where the SMRs were equidistant from the axis of rotation and the LT and rotation stage were in the same plane. Thus, the conditions did not identically match the experiments.

We performed similar simulations for the mirror-based approach. Again, for this set of simulations, we considered an idealized case where the LT and the rotation stage were in the same plane. In this case, the two parameters we could change were the distance of the LT to the rotation stage (*D_OA_*) and the distance of the target to the rotation stage (*D_OM_*; see Figure 3). Using the same values for uncertainty in the range and angles as discussed earlier, we calculated the uncertainty in the angle for a variety of cases and plotted the results in Figure 14b. These simulations were only performed for LT position A in Figure 4 and, therefore, the uncertainties shown in Figure 14b are the average of the uncertainties for the three angles (22.5°, 45°, and 67.5°). Decreasing the distance of the LT to the rotation stage and increasing the distance of the target to the rotation stage resulted in a smaller angle uncertainty. The solid black vertical and horizontal lines in the figure capture the situation encountered in our experiment, where *D_OM_* = 1500 mm, *D_OA_* = 1800 mm, and uncertainty was 0.4″.

## 5. Discussion

**Figure 15:**
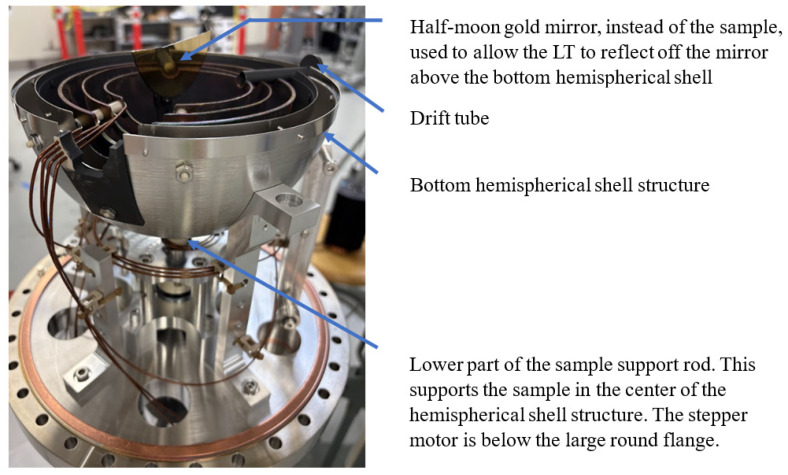
The setup for the calibration of the sample angle positioning system of the RFA.

While the focus of this paper was on a technique to calibrate angular positioning error using an LT, we briefly describe the RFA setup, which was the motivation for this work. The setup consisted of two sets of hemispherical shell structures welded together, a drift tube to introduce the electron beam through the shell structure, and a sample support rod with the sample at one end and connected to a stepper motor at the other end. Portions of the RFA are shown in Figure 15. The sample was replaced with a half-moon gold mirror for angle calibration using the LT.

**Figure 16:**
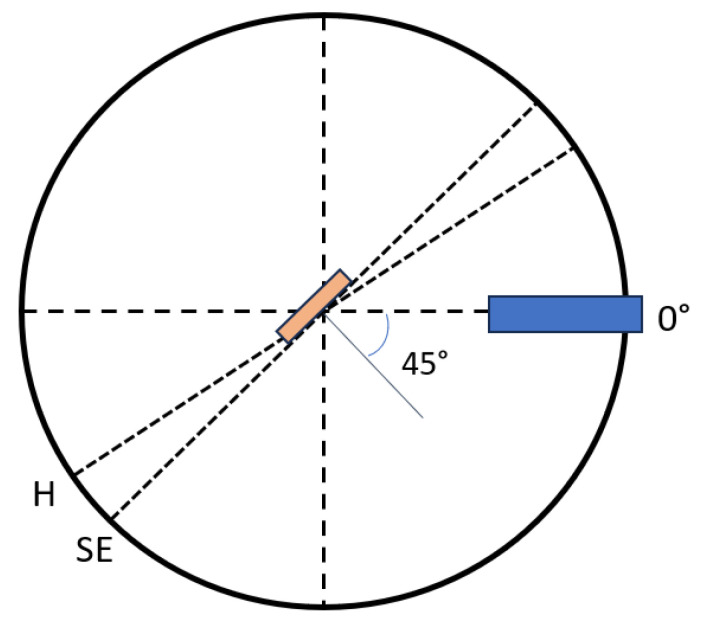
Schematic view of the sample positioning in the RFA. SE—sample exchange; H—home.

The stepper motor rotated at 1.8 degrees per step, resulting in 200 steps per revolution. Micro-stepping divided these 200 motor steps into smaller increments, enhancing smoothness and resolution. The stepper motor’s maximum resolution was 51,200 micro-steps per revolution. All motion was programmed in micro-steps per second. The stepper motor was equipped with an internal magnetic encoder, allowing it to return to a fiducial index mark position consistently. In the RFA, the stepper motor was used to rotate the sample support rod placed at the center of the shell structure to a specified angle. The sample angle was defined as the angle between the drift tube and the normal vector to the plane of the sample (see Figure 16). When the sample was aligned with the drift tube, it was in a position of normal incidence, corresponding to a zero-degree angle (0 steps on the stepper motor). The home position of the sample was set just beyond the sample exchange position, which was defined as 45° in the positive direction from the zero position, as illustrated in Figure 16. The sample was designed to be rotated from this home position to a sample position corresponding to 90° in the negative direction. This operational range was limited by switches that were connected to the stepper motor to prevent damage to the Kapton insulated wires leading to the sample and the sample support rod.

The calibration of the sample angle positioning system for the RFA was carried out following the methodology described in this study. A half-moon gold mirror sample was utilized for the measurements, which allowed the LT to reflect off the mirror above the bottom hemispherical shell structure, as shown in Figure 15. The angle, relative to the zero position, was measured by the LT within a sample angle range of [+20° to −90°]. The LT was positioned approximately in the middle of the range at −35°. Each specific angle corresponded to a distinct number of micro-steps on the stepper motor. Measurements were taken by executing a sweeping motion from one angle to another, directing the stepper motor to move a predetermined number of steps associated with each sample angle position. After conducting three repeated sweep measurements, a lookup table (LUT) was created. This table consisted of two columns: the number of steps and the angles measured by the LT, averaged over the three trials. The resulting LUT allowed for the positioning of the sample at any desired angle with an uncertainty smaller than 0.1° by commanding the stepper motor to move the adjusted number of steps, which was determined through interpolation of the LUT. Details on the RFA setup, the angle calibration data, and the uncertainty budget for the angles will be presented in a future publication.

The technique we have described in this paper does suffer from some limitations. Sufficient space around the rotation stage is necessary to install an LT and registration nests. If a plane mirror cannot be mounted on the rotation stage, clearly, this technique will not be applicable. The LT must be calibrated and meet its own specifications so that uncertainty estimated for angle measurements is reliable.

## 6. Conclusions

Measuring the angular positioning errors of a rotation stage in situ without modifications to underlying hardware is a commonly faced problem. This problem is even more challenging when there are physical constraints imposed by the setup conditions. In response to such a need encountered here at NIST, we developed a novel technique to measure angular positioning errors using an LT and a small plane mirror mounted on the rotation stage. At each angular position, we performed two LT measurements—a direct measurement to an SMR and an indirect measurement to the same SMR after reflection off the mirror. These measurements provided a simple and elegant way to calculate the direction of the normal vector of the plane mirror and, therefore, the angle between positions. We first validated our technique using a calibrated angle polygon, where the error in our LT mirror-based measurement was 0.16″. We then demonstrated the application of this technique using a precision spindle (whose angular positioning errors were calibrated using a Renishaw XR-20), where we recorded errors of about 0.5″ for the registration approach and about 0.7″ for the stitching approach. We also compared this mirror-based approach to a direct approach using two SMRs mounted on a bar, where we observed larger errors, about 1.7″. Our initial estimates for uncertainty (*k* = 2) were about 0.4″ for the mirror-based registration approach, about 0.4″ to 0.9″ for the mirror-based stitching approach (uncertainty depends on the angle), and about 2.4″ to 3″ for the direct approach (uncertainty depends on the angle).

There are additional steps we could take to reduce the uncertainty in our LT mirror-based measurements, such as by averaging the LT measurement from four angular positions (rotations about the azimuth) to reduce the effect of LT systematic errors. Preliminary measurements on polygons indicated that averaging multiple LT measurements would allow us to measure the angle at an uncertainty of about 0.1″. Performing high-accuracy angle measurements on calibrated polygons is a topic for future exploration.

## Figures and Tables

**Table 1:** Uncertainty and observed errors in seconds.

Method	Uncertainty, U (*k* = 2)	Observed Error Range	
	(Depends on Angle)	Minimum	Maximum
Direct	2.4 to 3	−1.7	+0.6
Mirror Stitching	0.4 to 0.9	−0.3	+0.7
Mirror Registration	0.4 to 0.5	−0.4	+0.5

## Data Availability

Data are contained within the article.
